# Supplementary material for: From sequence to enzyme mechanism using multi-label machine learning
Source: BMC Bioinformatics. 2014 May 19;15:150. doi: 10.1186/1471-2105-15-150 (PMC4229970; doi:10.1186/1471-2105-15-150)
Supplement: Additional file 2 — Java code of ml2db. Additional file ml2db_code.tar.gz contains the Java source code to run the multi-label machine learning experiments and save the results to database. The code’s Javadoc is included. [file 1471-2105-15-150-S2.zip › additional file 2/ml2db/ml2db/doc/mulan/classifier/transformation/MultiLabelStacking.html]

MultiLabelStacking


JavaScript is disabled on your browser.


- Overview
- Package
- Class
- Use
- Tree
- Deprecated
- Index
- Help

- Prev Class
- Next Class

- Frames
- No Frames

- All Classes

- Summary:
- Nested |
- Field |
- Constr |
- Method

- Detail:
- Field |
- Constr |
- Method


mulan.classifier.transformation

## Class MultiLabelStacking

- java.lang.Object
- - mulan.classifier.MultiLabelLearnerBase
  - - mulan.classifier.transformation.TransformationBasedMultiLabelLearner
    - - mulan.classifier.transformation.MultiLabelStacking

- All Implemented Interfaces:
  :   java.io.Serializable, MultiLabelLearner, weka.core.TechnicalInformationHandler

  ---

    

  ```
  public class MultiLabelStacking
  extends TransformationBasedMultiLabelLearner
  implements java.io.Serializable
  ```

  This class is an implementation of the (BR)^2 or Multi-Label stacking method.

  Version:
  :   2012.02.07

  Author:
  :   Eleftherios Spyromitros-Xioufis (espyromi@csd.auth.gr)

  See Also:
  :   Serialized Form

- - ### Constructor Summary

    Constructors

    Constructor and Description

    `MultiLabelStacking()`

    An empty constructor

    `MultiLabelStacking(weka.classifiers.Classifier baseClassifier,
    weka.classifiers.Classifier metaClassifier)`

    A constructor with 2 arguments
  - ### Method Summary

    Methods

    | Modifier and Type | Method and Description |
    | --- | --- |
    | `void` | `buildBaseLevel(MultiLabelInstances trainingSet)` Builds the base-level classifiers. |
    | `void` | `buildBaseLevelKNN(MultiLabelInstances trainingSet)` Used only in case of a kNN base classifier. |
    | `void` | `buildMetaLevel()` Builds the ensemble of meta-level classifiers. |
    | `weka.core.TechnicalInformation` | `getTechnicalInformation()` Returns an instance of a TechnicalInformation object, containing detailed information about the technical background of this class, e.g., paper reference or book this class is based on. |
    | `int` | `getTopkCorrelated()` Returns top k correlated labels |
    | `java.lang.String` | `globalInfo()` Returns a string describing the classifier. |
    | `void` | `initializeMetaLevel(MultiLabelInstances dataSet, weka.classifiers.Classifier metaClassifier, boolean includeAttrs, double metaPercentage, weka.attributeSelection.ASEvaluation eval)` Initializes all the parameters used in the meta-level. |
    | `void` | `saveObject(java.lang.String filename)` Saves a `MultiLabelStacking` object in a file |
    | `void` | `setEval(weka.attributeSelection.ASEvaluation eval)` Sets the attribute selection evaluation class |
    | `void` | `setIncludeAttrs(boolean includeAttrs)` Sets the value of includeAttrs |
    | `void` | `setMetaAlgorithm(weka.classifiers.Classifier metaClassifier)` Sets the type of the meta classifier and initializes the ensemble |
    | `void` | `setMetaPercentage(double metaPercentage)` Sets the value of metaPercentage |
    | `void` | `setNormalize(boolean normalize)` Sets the value of normalize |
    | `void` | `setPartialBuild(boolean partialBuild)` sets the value for partialBuild |

    - ### Methods inherited from class mulan.classifier.transformation.TransformationBasedMultiLabelLearner

      `getBaseClassifier`
    - ### Methods inherited from class mulan.classifier.MultiLabelLearnerBase

      `build, getDebug, isUpdatable, makeCopy, makePrediction, setDebug`
    - ### Methods inherited from class java.lang.Object

      `equals, getClass, hashCode, notify, notifyAll, toString, wait, wait, wait`

- - ### Constructor Detail


    - #### MultiLabelStacking

      ```
      public MultiLabelStacking()
      ```

      An empty constructor


    - #### MultiLabelStacking

      ```
      public MultiLabelStacking(weka.classifiers.Classifier baseClassifier,
                        weka.classifiers.Classifier metaClassifier)
      ```

      A constructor with 2 arguments

      Parameters:
      :   `baseClassifier` - the classifier used in the base-level
      :   `metaClassifier` - the classifier used in the meta-level
  - ### Method Detail


    - #### globalInfo

      ```
      public java.lang.String globalInfo()
      ```

      Returns a string describing the classifier.

      **Overrides:**
      :   `globalInfo` in class `TransformationBasedMultiLabelLearner`

      Returns:
      :   a string description of the classifier


    - #### getTechnicalInformation

      ```
      public weka.core.TechnicalInformation getTechnicalInformation()
      ```

      **Description copied from class: `TransformationBasedMultiLabelLearner`**

      Returns an instance of a TechnicalInformation object, containing detailed
      information about the technical background of this class, e.g., paper
      reference or book this class is based on.

      **Specified by:**
      :   `getTechnicalInformation` in interface `weka.core.TechnicalInformationHandler`

      **Overrides:**
      :   `getTechnicalInformation` in class `TransformationBasedMultiLabelLearner`

      Returns:
      :   the technical information about this class


    - #### initializeMetaLevel

      ```
      public void initializeMetaLevel(MultiLabelInstances dataSet,
                             weka.classifiers.Classifier metaClassifier,
                             boolean includeAttrs,
                             double metaPercentage,
                             weka.attributeSelection.ASEvaluation eval)
                               throws java.lang.Exception
      ```

      Initializes all the parameters used in the meta-level. Calculates the
      correlated labels if meta-level pruning is applied.

      Parameters:
      :   `dataSet` -
      :   `metaClassifier` -
      :   `includeAttrs` -
      :   `metaPercentage` -
      :   `eval` -

      Throws:
      :   `java.lang.Exception`


    - #### buildBaseLevel

      ```
      public void buildBaseLevel(MultiLabelInstances trainingSet)
                          throws java.lang.Exception
      ```

      Builds the base-level classifiers. Their predictions are gathered in the
      baseLevelPredictions member

      Parameters:
      :   `trainingSet` -

      Throws:
      :   `java.lang.Exception`


    - #### buildMetaLevel

      ```
      public void buildMetaLevel()
                          throws java.lang.Exception
      ```

      Builds the ensemble of meta-level classifiers.

      Throws:
      :   `java.lang.Exception`


    - #### buildBaseLevelKNN

      ```
      public void buildBaseLevelKNN(MultiLabelInstances trainingSet)
                             throws java.lang.Exception
      ```

      Used only in case of a kNN base classifier.

      Parameters:
      :   `trainingSet` -

      Throws:
      :   `java.lang.Exception`


    - #### saveObject

      ```
      public void saveObject(java.lang.String filename)
      ```

      Saves a `MultiLabelStacking` object in a file

      Parameters:
      :   `filename` -


    - #### setNormalize

      ```
      public void setNormalize(boolean normalize)
      ```

      Sets the value of normalize

      Parameters:
      :   `normalize` -


    - #### setIncludeAttrs

      ```
      public void setIncludeAttrs(boolean includeAttrs)
      ```

      Sets the value of includeAttrs

      Parameters:
      :   `includeAttrs` -


    - #### setMetaPercentage

      ```
      public void setMetaPercentage(double metaPercentage)
      ```

      Sets the value of metaPercentage

      Parameters:
      :   `metaPercentage` -


    - #### setEval

      ```
      public void setEval(weka.attributeSelection.ASEvaluation eval)
      ```

      Sets the attribute selection evaluation class

      Parameters:
      :   `eval` -


    - #### setMetaAlgorithm

      ```
      public void setMetaAlgorithm(weka.classifiers.Classifier metaClassifier)
                            throws java.lang.Exception
      ```

      Sets the type of the meta classifier and initializes the ensemble

      Parameters:
      :   `metaClassifier` -

      Throws:
      :   `java.lang.Exception`


    - #### setPartialBuild

      ```
      public void setPartialBuild(boolean partialBuild)
      ```

      sets the value for partialBuild

      Parameters:
      :   `partialBuild` -


    - #### getTopkCorrelated

      ```
      public int getTopkCorrelated()
      ```

      Returns top k correlated labels

      Returns:
      :   top k correlated labels


- Overview
- Package
- Class
- Use
- Tree
- Deprecated
- Index
- Help

- Prev Class
- Next Class

- Frames
- No Frames

- All Classes

- Summary:
- Nested |
- Field |
- Constr |
- Method

- Detail:
- Field |
- Constr |
- Method
